# Editorial: The role of the gut microbiota on bone mass in health and disease, volume II

**DOI:** 10.3389/fendo.2026.1972898

**Published:** 2026-09-14

**Authors:** Sadiq Umar, Abdul Malik Tyagi

**Affiliations:** 1 Department of Oral Biology, College of Dentistry, University of Illinois, Chicago, IL, United States; 2 Department of Orthopedic Surgery, School of Medicine, Washington University in Saint Louis, Missouri, MO, United States

**Keywords:** gut microbiota, immune system, inflammation, osteoarthritis, osteoporosis

The gut microbiota plays an important role in human health through its effects on metabolism (1), immune regulation (2), intestinal barrier integrity (3), and endocrine signaling (4). Increasing evidence suggests that alterations in gut microbial composition and function influence bone formation (5, 6), bone resorption (7), mineral metabolism, and systemic inflammation, highlighting the emerging concept of an integrated gut–immune–metabolic–bone axis rather than isolated pathways (6). This concept has also expanded to osteoarthritis (8), metabolic liver disease (9), and medication-induced skeletal disorders, illustrating how gut-driven metabolic and inflammatory processes extend beyond bone to joint and liver health.

This Research Topic brings together eight manuscripts examining the relationships among gut microbiota, bone, joints, metabolism, and immunity. Beyond summarizing individual contributions, these manuscripts collectively emphasize several unifying mechanisms: microbial metabolites (such as SCFAs), intestinal barrier integrity, immune regulation, nutrient absorption, and endocrine signaling converge to influence skeletal homeostasis. Collectively, these contributions provide a broad perspective on the emerging gut–bone and gut–joint axes and their therapeutic potential. Several articles address the mechanisms linking the gut microbiota to bone homeostasis. Study from Gong et al. describe a multifaceted gut–bone network in which microbial metabolites influence osteoblast and osteoclast activity, while microbial regulation of immunity affects pathways such as RANKL/OPG. The gut microbiota may also regulate endocrine signaling, intestinal barrier integrity, and nutrient absorption. Similarly, Basak et al. in his review highlights the role of gut metabolites in the gut–immune–bone axis. Metabolic disturbances associated with obesity, fatty liver disease, and chronic inflammation can alter microbial diversity and intestinal integrity, potentially affecting the availability of calcium, vitamin D, phosphorus, magnesium, and other nutrients required for bone health. The authors also discuss postbiotics as potential regulators of intestinal integrity and systemic inflammation.

Additionally, Wang et al. emphasized the interaction between estrogen deficiency, gut dysbiosis, systemic inflammation, and osteoclast activation in postmenopausal osteoporosis. Microbial communities and metabolites, including short-chain fatty acids, may influence T cells, B cells, macrophages, and bone remodeling. However, while probiotics, prebiotics, and fecal microbiota transplantation show promise, these approaches remain largely investigational, and robust clinical evidence demonstrating improvements in bone mineral density or fracture risk is still limited.

The relationship between medications, gut microbiota, and skeletal health is addressed by (Martiniakova et al.) Several commonly used drugs, including glucocorticoids, aromatase inhibitors, proton pump inhibitors, antiretroviral drugs, antiepileptic drugs, antipsychotics, and antidepressants, can adversely affect bone while also altering the gut microbiota. However, direct evidence that medication-induced microbial changes mediate alterations in bone turnover, bone mineral density, or fracture risk remains limited. The authors therefore emphasize the need for prospective and interventional studies. Gu et al. further discussed therapeutic manipulation of the gut–bone axis through dietary modification, probiotics, prebiotics, and fecal microbiota transplantation, while highlighting emerging approaches such as nano-biomaterials, bacterial synthetic biology, and novel small molecules.

Gut microbiota may also influence joint disease. Sun et al. reviewed the evidence linking gut dysbiosis with osteoarthritis, focusing on obesity, hyperuricemia, intestinal permeability, lipopolysaccharide, and short-chain fatty acids as potential contributors to systemic inflammation and joint degeneration. This expands the conceptual framework of the gut–bone axis by demonstrating that similar microbial and inflammatory pathways may also influence joint health. Complementing this review, Zhou et al. presented original findings from 55 patients with osteoarthritis and 55 healthy controls using 16S rRNA sequencing and untargeted fecal metabolomics. Significant differences in microbial composition and metabolic pathways were identified, with associations between specific bacteria and metabolites such as indole-3-acetic acid, luteolin, and D-tryptophan. These findings support a potential gut–metabolism–immunity–joint connection and may provide opportunities for future biomarker development, although causality remains to be established.

Finally, Zhang et al. examined the relationship between metabolic dysfunction-associated steatotic liver disease (MASLD) and osteoporosis. Both conditions share metabolic syndrome, insulin resistance, chronic inflammation, and altered intestinal permeability. Gut microbial dysregulation may connect hepatic and skeletal disease through microbial metabolites and systemic inflammatory signaling, broadening the scope of the Research Topic by showing how gut-driven metabolic dysfunction can influence multiple organ systems, including bone.

Taken together, these manuscripts reinforce the concept that gut microbiota is an important component of the interconnected network regulating bone and joint health. Microbial metabolites, intestinal barrier function, immune regulation, inflammation, nutrient absorption, and endocrine signaling emerge as recurring mechanisms across different conditions. However, much of the human evidence remains observational, and associations between microbial taxa and skeletal or joint phenotypes do not establish causality. This distinction is essential when interpreting mechanistic models and therapeutic implications. Future research should therefore combine longitudinal microbiome and metabolomic analyses with mechanistic studies and well-designed clinical trials to determine whether microbiota-directed interventions can improve clinically meaningful outcomes.
